# Duration of contact sports play associated with aberrant DNA methylation in human frontal cortex

**DOI:** 10.21203/rs.3.rs-7861173/v1

**Published:** 2025-12-01

**Authors:** Thor Stein, Kerry Breen, Nurgul Aytan, Samantha Hawkins, Raymond Nicks, Victor Alvarez, Jesse Mez, Jan Blusztajn, Jonathan Cherry, Ann McKee, Honghuang Lin

**Affiliations:** Boston University School of Medicine; Boston Chobanian & Avedisian University School of Medicine; Boston Chobanian & Avedisian University School of Medicine; Boston Chobanian & Avedisian University School of Medicine; Boston Chobanian & Avedisian University School of Medicine; Boston Chobanian & Avedisian University School of Medicine; Boston University School of Medicine; Boston University School of Medicine; Boston University School of Medicine; UMass Chan Medical School

## Abstract

Repetitive head impacts (RHI), primarily through contact sports play and military service, are a recognized risk factor for cognitive and behavioral symptoms, as well as progressive neurodegenerative diseases such as chronic traumatic encephalopathy. While altered DNA methylation has been linked to environmental exposures and neurodegeneration, its association with RHI remains unknown. In this study, we investigated whether duration of contact sports play in a community-based aging cohort is associated with altered DNA methylation patterns. Reduced representation bisulfite sequencing on human dorsolateral frontal cortex identified 461 genome-wide significant CpG sites associated with duration of contact sports play, spanning 13 genes of which the majority were hypomethylated. The hypomethylation pattern was largely replicated in an independent cohort. Notably, CAMK2B, B4GALT6, and TLR2, were hypomethylated and upregulated in the cortical sulcus of the DLFC in individuals with RHI exposure from contact sports. Furthermore, alterations of CAMK2B and B4GALT6 were observed in CTE cases. Together, these findings reveal distinct, region-specific epigenetic changes associated with contact sports exposure and provide new insights into the molecular mechanisms underlying RHI-related sequalae.

## INTRODUCTION

Millions of individuals are exposed to repetitive head impacts (RHI) every year, through contact sports, military service, or domestic violence. In contact sports, such as American football, athletes may sustain thousands of impacts per year, accumulating over multiple years.^1, 2^ Exposure to cumulative RHI can lead to severe cognitive and behavioral symptoms.^3–5^ RHI includes both clinically diagnosed concussions and non-concussive impacts, many of which go undocumented, making RHI difficult to quantify. As a result, duration of play (years) is frequently used as a proxy for cumulative RHI.^6, 7^ RHI is also the primary risk factor for development of chronic traumatic encephalopathy (CTE), a neurodegenerative tauopathy characterized by a unique distribution of hyperphosphorylated tau depositions within neurons and processes concentrated within cerebral sulci and surrounding small blood vessels.^6, 8^ The clinical manifestations of CTE, such as cognitive decline, behavioral changes and mood disturbances, are progressive and often appear years or decades after players have retired.^2, 9, 10^ Notably, the sulcal depths of the dorsolateral frontal cortex (DLFC) show early and regionally selective vulnerability following RHI^11–13^, but the molecular mechanisms involved remain poorly understood.

DNA methylation is one of the most extensively studied epigenetic modifications, involving the addition of a methyl group to a nucleotide—most commonly a cytosine that precedes a guanine, referred to as a CpG site. DNA methylation is influenced by various environmental exposures, such as smoking, air pollution, and psychological stress.^14–17^ Repetitive head impacts, as a form of mechanical environmental stress, may likewise alter DNA methylation patterns. In fact, preclinical models of mild or blast-related TBI have demonstrated sustained molecular alterations, including changes in DNA methylation, months after injury.^18^ However, no study to date has investigated the association between duration of play and DNA methylation alterations in humans. Over the past decade, a growing body of evidence has linked DNA methylation to multiple neurological diseases and disorders, including Parkinson’s disease, Alzheimer’s disease, and post-traumatic stress disorder.^19–25^ Given that DNA methylation is a stable epigenetic modification that regulates gene expression and is modulated by environmental factors, it represents a promising molecular marker for investigating the effects of RHI in human postmortem tissue.

In this study, we tested the hypothesis that duration of contact sports play is associated with aberrant DNA methylation patterns that drive downstream transcriptomic and proteomic alternations in human postmortem DLFC tissue from a community-based cohort. Leveraging this community-based cohort allows us to investigate the effects of low level RHI exposure, providing a unique opportunity to detect molecular changes independent of overt pathology. Reduced representation bisulfite sequencing was used to evaluate the association between DNA methylation at single-nucleotide resolution and duration of contact sports play. Further, top differentially methylated genes were investigated for transcript alterations using fluorescent *in situ* hybridization and protein alterations with multiplex immunofluorescence.

## METHODS

### Study cohort and case selection

The Framingham Heart Study (FHS) is a longitudinal observational cohort that follows multiple generations of participants, collecting biological and lifestyle data to study cardiovascular, neurological, and other diseases.^26^ As part of this effort, some participants elect to donate their brains for research. Inclusion criteria for the present study were based on frozen tissue availability, DNA quality, and contact sport exposure information. Due to no females having documented contact sports exposure in the FHS brain bank, females were excluded. Further exclusion criteria included hanging, drug overdose, or gunshot wound to the head as the cause of death as it may compromise tissue quality. For the discovery cohort, FHS human postmortem brain tissue obtained from 2/2000 to 7/2018 were queried. During this period, a total of 210 brain donations were available, of which 89 cases met our inclusion criteria. For the replication cohort, we queried FHS brain donations between 9/2014 and 9/2021. Among 34 donors, 13 met inclusion criteria. As none of these FHS cases had documented RHI exposure, we included additional cases from the Understanding Neurological Injury and Traumatic Encephalopathy (UNITE) brain bank between 7/2009 and 9/2018. There were a total of 404 brain donations meeting our inclusion/exclusion criteria during this period. Briefly, inclusion in the UNITE brain bank requires a history of RHI (e.g., from contact sports, military service, physical violence, or other sources) and tissue quality sufficient for neuropathological diagnosis through immunohistochemistry.^27^ To more closely match the FHS cohort, those with CTE were excluded and the remaining donors were age-matched to the FHS controls yielding an additional 30 UNITE donors with RHI history for a total of 43 brain donors in the replication cohort.

For our follow-up studies using fluorescent *in situ* hybridization (FISH) and multiplex immunofluorescence (mIF), cases were selected based upon tissue availability for the RHI cases included in the RRBS discovery set. We then selected age- and neurodegenerative matched control cases (individuals with 0 years of contact sports) for the available RHI cases. For fresh frozen tissue for FISH a total of 11 RHI cases were available, and these were matched to 15 control cases. For formalin fixed paraffin embedded tissue for mIF a total of 17 RHI cases were available, and these were matched to 10 control cases. An additional 8 representative CTE cases from UNITE were included to examine proteins of interest in disease severity, cases were stratified into low (Stage I–II) and high (Stage III–IV) CTE pathology based on established staging criteria.^2^ Consent for use of the tissue for research purposes was obtained from the legal health care proxy by the respective institutions.

Institutional review board approval for brain donation and informed consent for research was obtained through the Boston University Alzheimer’s Disease and CTE Center, Human Subjects Institutional Review Board of the Boston University Medical Campus, and VA Bedford Healthcare System, MA.

### DNA extraction and preparation for reduced representation bisulfite sequencing (RRBS)

**Figure 1:**
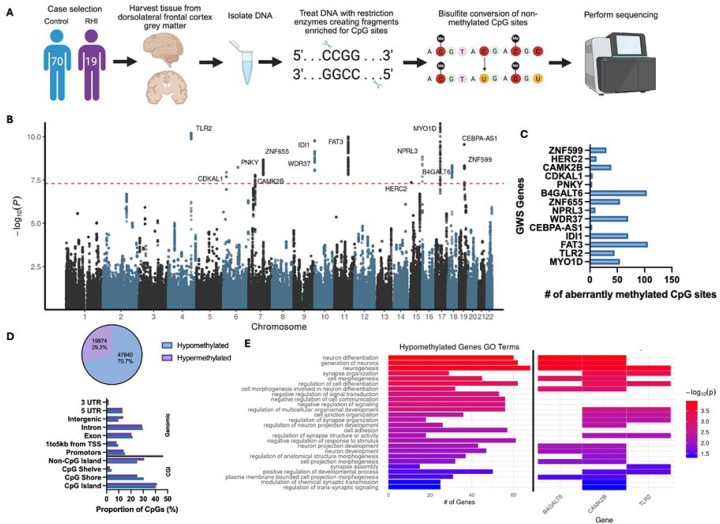
Aberrant DNA methylation associated with duration of contact sports play. (A) Diagram depicting RRBS workflow. Created in BioRender. Breen, K. (2025) https://BioRender.com/czxsqhy (B) Manhattan plot displaying the association between aberrant DNA methylation and duration of contact sports play (years). Genome-wide significance line (p < 5 × 10^−8^) is denoted by the red line. (C) Bar plot showing the number of aberrantly methylated CpG sites in each GWS DMR. (D) Pie chart showing percent of hypo- and hypermethylated CpG sites (top) and bar plot displaying genomic and CpG island (CGI) annotations of CpG sites (bottom). (E) Gene ontology analysis on hypomethylated gene list (left) and displaying which GO terms included *CAMK2B*, *B4GALT6*, and *TLR2* (right). D-E performed with genes/CpG sites with a p<0.05.

Fresh frozen brain tissue was collected from the gray matter dorsolateral frontal cortex of each donor. The tissue was processed and cleaned of white matter and blood, then was harvested to include 500ng of grey matter tissue. DNA was extracted using the Promega Maxwell RSC Tissue DNA kit following their protocols. Sample concentration and quality was measured using a Nanodrop and all samples had a 260/280 and 260/230 OD ratios greater than 1.7. Reduced representation bisulfite sequencing was performed by Zymo Research (Orange, CA, USA) using their Classic RRBS service, which interrogates DNA methylation of > 1.5 million sites at 5–10x minimum coverage (Fig. 1A).

### RRBS data preprocessing and analysis

The sequencing data underwent rigorous quality control, including adapter trimming, exclusion of CpG sites with fewer than 10 reads, and removal of CpG sites profiled in fewer than 10 samples. Methylation levels were estimated using BSmooth.^28^ Reference genome alignment was performed with GRCh37/hg19. A total of 1,914,000 CpG sites passed quality control and were included in the analysis. Assuming one million independent CpG sites, the Bonferroni corrected genome-wide significance threshold was set to 5×10^−8^. Linear regression models were applied to assess the association between duration of contact sports play and CpG site-specific methylation levels derived from RRBS. Duration of contact sports play was a continuos variable of concurrent years of play ranging from 0–19 years. Models were adjusted for potential confounders, including age at death and smoking history. Differentially methylated regions (DMRs) were defined as regions ≥100 base pairs in length, containing at least three significant CpG sites, with over 80% of CpG sites in the region reaching significance.

### Gene ontology analysis

Gene ontology analysis was performed using g:Profiler with the g:GOST functional profiling tool.^29^ This database integrates information from Ensembl, Gene ontology (GO molecular function, GO biological process, GO cellular component, and biological pathways), KEGG, Reactome, and Wikipathys.

### Single molecule fluorescent mRNA *in situ* hybridization

Fresh frozen DLFC tissue containing a sulcus and gyrus was embedded in Optimal Cutting Temperature medium (Sakura Tissue-Tek) and was equilibrated to cryostat temperature (-20⁰ C) before cutting. Chuck temperature was set to −12⁰/ −10⁰C for optimal cutting conditions. Tissue sections were cut at a thickness of 16 μm onto Fisher SuperFrost slides. Sections were fixed in cold 10% Neutral Buffered Formalin (4°C) for 60 minutes, followed by dehydration in 50%, 70%, and two changes of 100% ethanol, each for 5 minutes at room temperature. FISH was performed using RNAScope multiplex assay kits (Advanced Cell Diagnostics) optimized on the Leica BOND Rx automated slide staining system (Leica Biosystems). Slides were pretreated with protease at room temperature for 15 minutes. Opal TSA dyes were used for visualization at a concentration of 1:500. A positive and negative control probe was run for each block before staining with targeted probes. Slides were coverslipped with ProLong Gold Antifade mounting medium (Invitrogen). The slides were imaged at 40x on a PhenoImager 2.0 software whole-slide scanner and were spectrally unmixed during scanning.

### Multiplex immunofluorescence staining

Formalin fixed, paraffin embedded tissue was obtained from the DLFC and sectioned on a microtome at 10mm. The slides were dried, baked, dewaxed, and rehydrated prior to staining. Epitope retrieval was optimized for each antibody to be at pH 6 or 9 buffer for 15 minutes in the microwave. Sections were blocked for 30 minutes at room temperature with 3% donkey serum and then incubated with primary antibodies for 1 hour at room temperature or overnight at 4°C. The primary antibodies were CAMK2B (Abcam, 1:500), B4GALT6 (Proteintech 1:100), TLR2 (R&D Systems, 1:1000 overnight), HuC/HuD (Invitrogen, 1:500), IBA1 (Wako, 1:500), GFAP (Biolegend, 1:750), and CD3 (Proteintech, 1:200). Secondary HRP-conjugated antibodies were incubated for 30 minutes at room temperature, followed by Opal TSA dyes for 10 minutes and incubated with DAPI for 5 minutes to label cell nuclei. Slides were coverslipped with ProLong Gold Antifade mounting medium (Invitrogen). The slides were imaged at 20x on a PhenoImager 2.0 software whole-slide scanner and were spectrally unmixed during scanning.

### Image analysis

Analyses of FISH was performed in Indica Labs HALO using the FISH v3.2.3 algorithm. Thresholds for FISH probe positivity, based upon size, intensity, and colocalization with DAPI, were set manually for each probe (*CAMK2B, B4GALT6, TLR2*) and kept consistent across samples. Analyses of immunofluorescence staining was performed in Indica Labs HALO using the Highplex v4.3.4 algorithm and HALO AI nuclei segmentation classifier. The classifier was given examples of brain parenchyma annotated for each protein of interest (CAMK2B and B4GALT6) which were considered positive based on intensity and colocalization with DAPI. Each classifier was given over 1000 examples and was allowed to iterate over 80,000 times. AI classifier was used to help with identifying true positives in cases with higher background staining. Thresholds for antibody positivity, based upon intensity (nuclear or membrane) and the % completeness of the nuclear or membrane positivity, were manually set for each antibody and kept consistent across samples. For FISH and IF sections, the depth of the cortical sulcus was defined and annotated as the area 2mm from the bottom of the sulcus including both sides and the gyrus was defined as a third of the adjacent gyrus to the sulcus analyzed. Quantifications in the sulcus and gyrus were normalized to area analyzed.

### Statistical analysis

Descriptive statistics were used to summarize demographic and clinical characteristics. Group comparisons between individuals with repetitive head impact (RHI) exposure and controls were conducted using two-tailed independent sample t-tests for continuous variables and chi-squared tests for categorical variables.

Transcript quantification from FISH and protein expression from mIF were log-transformed to improve normality. Comparisons between RHI-exposed and control groups were performed using unpaired t tests with Welch’s correction on the transformed data. General linear regression models with binned years of play was used to evaluate the assocation between increaing years of play and transcript quantifications. For comparisons across CTE stage groups (control, low CTE, and high CTE), one-way analysis of variance (ANOVA) was performed, followed by Sidak’s post hoc multiple comparisons test. All statistical analyses were performed using R (version 2024.12.0) and/or Prism (GraphPad, version 10.5.0). Statistical significance was set as p < 0.05 unless otherwise specified.

## RESULTS

### DNA methylation alterations associated with duration of contact sports play implicated in neurogenesisand synaptic organization pathways

A total of 89 samples were included in the discovery set with a mean age at death of 84 years, of which 19 (21.3%) played contact sports (e.g. football and hockey) with an average of 6 years of play. Notably, the control and RHI-exposed groups did not differ with respect to age at death, race, neurodegenerative pathology, or cerebrovascular pathology. Sample statistics for the discovery set are shown in Table 1. A total of 461 CpG sites reached genome-wide significance (GWS) for their association with duration of contact sports play (Fig. 1B, Supplementary Table 1). These CpG sites span a total of 13 genes, all of which contain differentially methylated regions (Fig. 1C). Of the 13 genes, 9 showed hypomethylation associated with increasing duration of play. Further, querying all CpG sites with a p-value less than 0.05 (n=67714), 70.7% CpG site were hypomethylated and 29.3% CpG sites were hypermethylated with increasing duration of play (Fig. 1D, Supplementary Table 1). Functional annotation of the duration of contact sports associated CpG sites showed that most of the aberrant methylation is occurring in introns and CpG islands (Fig. 1D). Of our GWS methylated genes, *B4GALT6*, *IDI1*, *WDR37*, *ZNF599*, and *ZNF655* have multiple aberrantly methylated CpG sites within promotors. Further, *TLR2* has aberrantly methylated CpG sites that are 1 to 5 kilobases (kb) away from a transcriptional start site (TSS). In general, the majority of the GWS methylation is occurring within CpG islands and shores.

Gene ontology analyses on all hypomethylated genes with a p<0.05 associated with duration of contact sports play revealed “neurogenesis”, “neuron differentiation”, and “synapse organization” as top terms (Fig. 1E). Notably, of the 27 ontology terms, *CAMK2B* was contained in the most (20 terms), followed by *B4GALT6* and *TLR2* (9 terms each, Fig. 1E).

### Replication and sensitivity analyses

A total of 43 samples were included in the replication group with a mean age of death of 84 years, of which 30 (69.8%) played contact sports with an average of 9 years of play. *MYO1D, TLR2, FAT3, IDI1, CEBPA-AS1, WDR37, ZNF655, B4GALT6, CAMK2B,* and *ZNF599* demonstrated consistent directions of methylation change with duration of play in the replication group (Supplementary Table 2). Among these, only *B4GALT6* remained statistically significant in the replication cohort (Top CpG: Chr18:29265538, p=0.00226, Supplementary Table 2).

To confirm that the GWS CpG sites from the discovery set were not driven by underlying neurodegenerative pathology, we conducted a sensitivity analysis on the 57 discovery set cases that had no neurodegenerative diagnoses (Alzheimer’s, Lewy body disease, frontal lobar degeneration).The GWS CpG sites from the discovery set remained significant, indicating that the results were not influenced by comorbid neurodegenerative diseases (Supplementary Table 3).

### *CAMK2B, B4GALT6,* and *TLR2 are* upregulated in the cortical sulcus in individuals with RHI exposure

**Figure 2:**
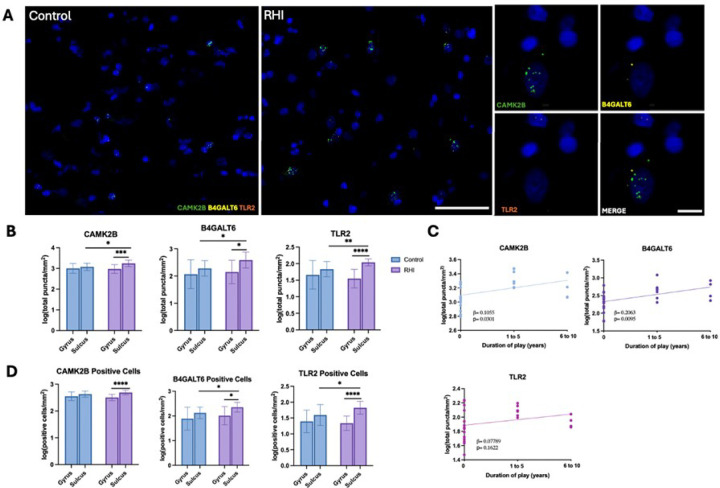
Increased cortical sulcus expression of *CAMK2B, B4GALT6* and *TLR2* in individuals with RHI exposure. (A) Representative images of *in situ*hybridization of *CAMK2B* (green), *B4GALT6* (yellow), and *TLR2*(orange). Left scale bar 50μm, right scale bar 10μm. (B) Bar plot of quantifications of *CAMK2B, B4GALT6*, and *TLR2* transcript copies/puncta. *p<0.05 **p<0.01 ***p<0.005 ****p<0.0005 (C) Scatter plot showing *CAMK2B, B4GALT6*and *TLR2* copies across duration of play groups. Dots depict individual samples; line represents general linear regression. (D) Bar plot of quantifications of *CAMK2B, B4GALT6*, and *TLR2* cellular density. *p<0.05 ****p<0.0005

*CAMK2B*, *B4GALT6, and TLR2* were the three GWS genes most frequently represented among the top GO terms and were therefore selected for follow-up gene expression analysis using FISH (Fig. 2A). Comparisons between RHI and control groups showed increased transcript copies of *CAMK2B* (p=0.0168), *B4GALT6* (p=0.0111), and *TLR2* (p=0.0052) in the cortical sulcus of RHI compared to controls, whereas no differences were observed in the cortical gyrus (*CAMK2B* p=0.4213, *B4GALT6* p=0.6626, *TLR2* p=0.4273, Fig. 2B). Within-group comparisons revealed that the RHI group had higher sulcal expression relative to gyral crest expression for all three genes (RHI: *CAMK2B* p=0.0026, *B4GALT6* p=0.0110, *TLR2* p=0.0001 and Controls: *CAMK2B* p=0.7091, *B4GALT6* p=0.1888, *TLR2* p=0.1778, Fig. 2B), a pattern not seen in controls (Fig. 2B). Linear regression analyses further demonstrated that *CAMK2B* (b=0.1055, p=0.0301) and *B4GALT6* (b=0.2063, p=0.0095) transcript levels in the cortical sulcus increased with binned years of contact sports play, while TLR2 (b=0.07789, p=0.1622) showed a positive but non-significant association (Fig. 2C). To distinguish whether these changes reflected altered expression within cells or an increased number of expressing cells, we quantified transcript-positive cell density. This analysis revealed a consistent pattern of higher positive cell density at the sulcus compared the gyrus within the RHI group for *CAMK2B* (p=0.0004)*, B4GALT6* (p=0.0156), and *TLR2* (p=0.0001, Fig. 2D). There was also significantly increased sulcal *B4GALT6* (p=0.0114) and *TLR2* (p=0.0398) positive cell density in RHI compared to controls (Fig. 2D).

### Loss of CAMK2B and B4GALT6 in the cortical sulcus in CTE

**Figure 3:**
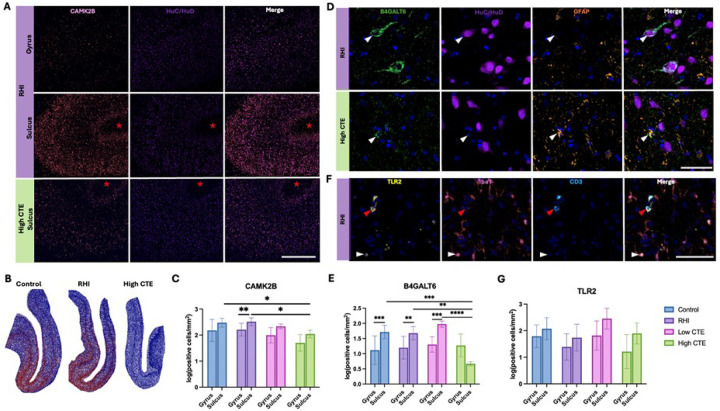
Cell-type localization and regional alterations of CAMK2B, B4GALT6, and TLR2 in control, RHI, and CTE. (A) Representative images of mIF of CAMK2B (yellow) and HuC/HuD (orange), red asterisks indicate depth of the sulcus. Scale bar 1mm. (B) Representative density heatmaps of CAMK2B+/DAPI (red) and CAMK2B-/DAPI (blue). (C) Bar plot of quantifications of CAMK2B positive cells. *p<0.05 **p<0.01 (D) Representative images of mIF of B4GALT6 (green), GFAP (orange), and HuC/HuD (red) at the depths of the cortical sulcus, solid arrow in RHI shows a B4GALT6+/HuC/HuD+ cell and solid arrow in high CTE shows a B4GALT6+/GFAP+ cell. Scale bar 50μm. (E) Bar plot of quantifications of B4GALT6 positive cells. **p<0.01 ***p<0.005 ****p<0.0001 (F) Representative images of mIF of TLR2 (orange), Iba1 (yellow), and CD3 (green) at the depths of the cortical sulcus, solid white arrow shows a TLR2+/Iba1+ cell and solid red arrow shows a TLR2+/CD3+ cell. Scale bar 100μm. (E) Bar plot of quantifications of TLR2 positive cells.

Immunofluorescence revealed that CAMK2B overlapped with the neuronal marker HuC/HuD (Fig. 3A). There was no significant difference in protein levels of CAMK2B between RHI and control cases; however, within the RHI group, there was significantly increased CAMK2B within the sulcus compared to the gyral crest (p=0.0068, Fig. 3B–C). Further, there was a significant decrease in CAMK2B in the cortical sulcus within High CTE compared to the RHI group (p=0.0101) and control group (p=0.0383, Fig. 3B–C). B4GALT6 expression was primarily neuronal (HuC/HuD) with some astrocytic (GFAP) expression (Fig. 3D). The protein level of B4GALT6 was increased in the sulcus compared to the gyrus within the control (p=0.0031), RHI (p=0.0090), and Low CTE (p=0.0037) groups (Fig. 3E). When comparing between groups High CTE had decreased sulcal B4GALT6 compared to all other groups (Controls p=0.0034, RHI p=0.0092, Low CTE p<0.0001, Fig 3E). Further, in High CTE there was no difference between sulcus vs gyral crest B4GALT6 (p=0.8906, Fig. 3E). TLR2 was primarily expressed in microglia (Iba1) with some T cell (CD3) expression (Fig. 3F). No significant differences in protein levels of TLR2 were observed across control, RHI, Low CTE, or High CTE groups (Fig. 3G).

## DISCUSSION

We conducted the first epigenetic study of contact sports exposure and identified novel differentially methylated CpG sites associated with the duration of contact sports play. Specifically, we identified 461 GWS CpG sites associated with duration of play, which spanned a total of 13 genes, including *MYO1D, TLR2, FAT3, IDI1, CEBPA-AS1, WDR37, NPRL3, ZNF655, B4GALT6, PNKY, CDKAL1, CAMK2B, HERC2, and ZNF599*. These aberrantly methylated genes are broadly involved in synaptic function and neurodevelopment (*CAMK2B, B4GALT6, FAT3, PNKY, WDR37*)^30–35^, neuroinflammation and immune response (*TLR2*)^36, 37^, cell signaling and metabolism (*NPRL3, IDI1, CDKAL1*)^38–40^, transcriptional regulation and genomic maintenance (*CEBPA-AS1, ZNF655, ZNF599, HERC2*)^41–43^ and cell structure and trafficking (*MYO1D*).^44^ Most of the CpG sites were hypomethylated with increasing duration of contact sports play. The direction of the methylation was replicated in an independent cohort of individuals with and without contact sports exposure. The aberrantly hypomethylated genes are involved in GO pathways of neurogenesis, neuron differentiation, and synapse organization, suggesting an adaptive response to injury. Consistent with their hypomethylation, we found increased transcript expression of *CAMK2B, B4GALT6,* and *TLR2* at the cortical sulcus between RHI and control, as well as within RHI comparing sulcus and gyrus. Finally, we observed reduced cellular densities of CAMK2B and B4GALT6 within the sulcus in high stage CTE, indicating potential downstream neurodegenerative consequences.

Our findings have three main implications in the field of neurotrauma and neuroepigenetics. First, even low levels of contact sports exposure in a community-based cohort are associated with long-lasting, coordinated DNA methylation changes detectable decades after exposure. Second, the cortical sulcus specificity of our findings supports the idea that these molecular changes are driven by mechanical injury resulting from RHI. Third, expression levels of CAMK2B and B4GALT6 change with the development of CTE. Collectively, these results identify a robust, sulcus-specific genetic signature of RHI exposure that may reflect an early adaptive response, which is ultimately lost or overwhelmed during disease progression.

Building on the finding that contact sports exposure—even at low levels within this community-based cohort—is associated with a coordinated change in DNA methylation detectable decades after exposure, studies in mild traumatic brain injury rat models have demonstrated that aberrant methylation patterns can persist for months after injury.^18^ Similarly, our results show genome-wide significant methylation changes in 13 genes associated with duration of contact sports play, indicating these epigenetic alterations are long-lasting and may represent a durable molecular signature of RHI. Furthermore, most of these changes reflect a hypomethylation signature in genes related to neurogenesis, neuron differentiation, and synapse organization. As DNA hypomethylation is generally associated with increased transcriptional activity, this epigenetic shift may promote greater gene expression. Upregulation of these pathways may represent an early compensatory response to neuronal damage. Previous methylation work with cellular resolution has shown most brain methylation changes occur in neurons.^45, 46^ Consistently, most of our implicated pathways relate to neuronal health and two of our three follow-up candidates were expressed and altered within neurons. In contrast, B4GALT6 was also expressed in GFAP+ astrocytes and TLR2 in Iba1+ microglia and CD3+ T cells, suggesting a broader involvement across cell types. Therefore, future work investigating methylation changes in glial and inflammatory cells is warranted. Overall, these findings highlight that long-lasting epigenetic alterations may act as molecular footprints of contact sports exposure and offer mechanistic insight into how such exposure predisposes individuals to developing symptoms or neuropathological changes.

As demonstrated by the cortical sulcus specificity of our findings, these molecular changes appear to be driven by mechanical injury resulting from RHI. Prior neuropathological studies demonstrate that the depths of the cortical sulcus are the earliest and most consistently affected regions in CTE, likely reflecting their heightened biomechanical vulnerability during trauma.^11, 12, 47, 48^ Additionally, longer contact sports duration is associated with neurodegenerative features such as decreased cortical thickness, especially within the sulcus and independent of tau pathology.^12, 48^ Although these relationships have typically been documented in cohorts with high RHI exposure (average 10–15 years of play), our results from a community-based cohort with lower exposure show aberrant hypomethylation associated with longer play duration and upregulation of key genes both in the RHI group compared to controls, and specifically at the cortical sulcus compared to the adjacent gyrus. These results indicate that even low levels of RHI exposure can induce sulcus-specific molecular changes, which may reflect an early and localized adaptive response to injury.

CAMK2B and B4GALT6 change with the development of CTE and are both critical for neuronal function and development. CAMK2B, a calcium-calmodulin dependent kinase, is essential for learning, memory, synaptic plasticity, and neurogenesis,^30, 49, 50^ and its dysregulation has been implicated in neurodevelopmental, psychiatric, and neurodegenerative disorders.^51, 52^ Notably, reduced CAMK2B transcript and protein expression is observed in Alzheimer’s disease, where it is associated with impaired synaptic function.^53–56^ *CAMK2B* is also a TDP-43 regulated gene that is reduced in multiple TDP-43 related diseases.^57–59^ B4GALT6, a key glycoprotein involved in the synthesis of lactosylceramide —the precursor for all gangliosides—plays an important role in neural development, extracellular matrix formation, myelination, and astrocyte activation during neuroinflammation.^31, 60, 61^ Our finding of reduced CAMK2B and B4GALT6 protein levels in high stage CTE suggests disruption of synaptic integrity and neuronal function may accompany advanced disease and cumulative RHI exposure. This molecular loss aligns with neuropathological findings of marked neuronal loss and cortical thinning at the sulcal depths in CTE, particularly among individuals with prolonged contact sports exposure, reinforcing the notion of sulcus-selective vulnerability in RHI-related neurodegeneration. Importantly, this decline in neuronal gene expression in CTE likely reflects a failure of the early compensatory molecular response to injury, which becomes diminished or maladaptive as neurodegenerative pathology accumulates and disease progresses.

Several limitations should be acknowledged. Although this represents the largest study to date examining methylation changes associated with contact sports exposure, the overall sample size remains modest. However, our analyses focused on differentially methylated regions rather than individual CpG sites, supporting the robustness of the findings. The inclusion of only male participants is another limitation, underscoring the need for future research to assess possible sex differences in the molecular response to RHI. Furthermore, our mIF experiments were constrained by tissue availability and by the number of cases with RHI exposure, which was intended to define spatial specificity. Expanding the sample size in future studies will improve the power to detect subtler molecular alterations and clarify cell-type–specific expression patterns.

Overall, these findings demonstrate that even low levels of contact sports play are associated with persistent molecular changes, underscoring the sensitivity of the human brain to cortical injury in the absence of overt neuropathology. Notably, these alterations are highly localized to the cortical sulcus, a region particularly venerable to mechanical strain during head trauma. By linking epigenetic dysregulation to region-specific vulnerability, this study provides a new perspective on how early molecular events triggered by repetitive head impacts may contribute to later neurodegeneration. These findings lay the groundwork for future mechanistic studies to define the role of epigenetic alterations in RHI-associated neurodegeneration and to support the development of molecular biomarkers for early detection and intervention.

## Supplementary Files

This is a list of supplementary files associated with this preprint. Click to download.

- SupplementaryTable1.xlsx
- SupplementaryTable2.xlsx
- SupplementaryTable3.xlsx

## Figures and Tables

**Table 1.** Demographic and pathological characteristics of the discovery set.

	Total (n=89)	Controls (n=70)	RHI (n=19)	p
**Age at death,** mean (SD),y	84 (10.2)	84 (10.0)	84 (11.4)	0.8898
**Years of play,** mean (SD), y, [range]	--	0	6.10 (3.98) [2–19]	--
**Football,** % (n)	14.6 (13)	--	68.4 (13)	--
Football years, mean (SD), y	--	--	4.14 (1.51)	--
**Hockey,** % (n)	6.74 (6)	--	31.6 (6)	--
Hockey years, mean (SD),y		--	8.71 (3.45)	--
**Race**, % (n)				0.6692
White	92.1 (82)	92.9 (65)	89.4 (17)	
Black/African American	1.12 (1)	1.43 (1)	0	
Other/missing	6.74 (6)	5.71 (4)	10.6 (2)	
**Educational level**, % (n)				0.2130
Some or high school diploma/GED	31.5 (28)	31.4 (22)	31.6 (6)	
Some college (no degree)	18.0 (16)	21.4 (15)	5.26 (1)	
College degree	50.6 (45)	47.2 (33)	63.1 (12)	
**Smoking history**, % (n)	36.0 (32)	34.3 (24)	42.1 (8)	0.5940
**Military History**, % (n)	31.5 (28)	28.6 (20)	42.1 (8)	0.2768
**Neurodegenerative pathology**,% (n)				
Alzheimer’s disease	28.1 (25)	28.6 (20)	26.3 (5)	0.9999
Frontotemporal lobar degeneration	8.99 (8)	10.0 (7)	5.26 (1)	0.9999
Lewy body disease (Limbic or neocortical)	11.2 (10)	10.0 (7)	15.8 (3)	0.4395
**Cerebrovascular pathology (moderate to severe),** % (n)				
Cerebral amyloid angiopathy	28.1 (25)	31.4 (22)	12.0 (3)	0.2527
Arteriosclerosis	58.4 (52)	70.0 (49)	68.4 (13)	0.9999
Atherosclerosis	42.7 (38)	40.0 (28)	52.6 (10)	0.4337
**Dementia**, % (n)	8.99 (8)	10.0 (7)	5.26 (1)	0.9999

Data are presented as mean (SD) for continuous variables and as % (n) for categorical variables.

## References

[1] Daneshvar DH, Nowinski CJ, McKee AC, Cantu RC. The epidemiology of sport-related concussion. Clin Sports Med. 2011;30(1):1–17, vii. doi: 10.1016/j.csm.2010.08.006. 21074078 PMC2987636

[2] McKee AC, Stein TD, Huber BR, Crary JF, Bieniek K, Dickson D, Alvarez VE, Cherry JD, Farrell K, Butler M, Uretsky M, Abdolmohammadi B, Alosco ML, Tripodis Y, Mez J, Daneshvar DH. Chronic traumatic encephalopathy (CTE): criteria for neuropathological diagnosis and relationship to repetitive head impacts. Acta Neuropathologica. 2023. doi: 10.1007/s00401-023-02540-w. PMC10020327 36759368

[3] Schaffert J, Datoc A, Sanders GD, Didehbani N, LoBue C, Cullum CM. Repetitive head-injury exposure and later-in-life cognitive and emotional outcomes among former collegiate football players: a CLEAATS investigation. Int Rev Psychiatry. 2024;36(3):233–42. Epub 20240514. doi:10.1080/09540261.2024.2352572. 39255023

[4] Alosco ML, Tripodis Y, Baucom ZH, Mez J, Stein TD, Martin B, Haller O, Conneely S, McClean M, Nosheny R, Mackin S, McKee AC, Weiner MW, Stern RA. Late contributions of repetitive head impacts and TBI to depression symptoms and cognition. Neurology. 2020;95(7):e793–e804. Epub 20200626. doi: 10.1212/wnl.0000000000010040. 32591472 PMC7605500

[5] Manley G, Gardner AJ, Schneider KJ, Guskiewicz KM, Bailes J, Cantu RC, Castellani RJ, Turner M, Jordan BD, Randolph C, Dvořák J, Hayden KA, Tator CH, McCrory P, Iverson GL. A systematic review of potential long-term effects of sport-related concussion. Br J Sports Med. 2017;51(12):969–77. Epub 20170428. doi: 10.1136/bjsports-2017-097791. 28455362 PMC5466926

[6] Mez J, Daneshvar DH, Abdolmohammadi B, Chua AS, Alosco ML, Kiernan PT, Evers L, Marshall L, Martin BM, Palmisano JN, Nowinski CJ, Mahar I, Cherry JD, Alvarez VE, Dwyer B, Huber BR, Stein TD, Goldstein LE, Katz DI, Cantu RC, Au R, Kowall NW, Stern RA, McClean MD, Weuve J, Tripodis Y, McKee AC. Duration of American Football Play and Chronic Traumatic Encephalopathy. Ann Neurol. 2020;87(1):116–31. Epub 20191123. doi: 10.1002/ana.25611. 31589352 PMC6973077

[7] Daneshvar DH, Nair ES, Baucom ZH, Rasch A, Abdolmohammadi B, Uretsky M, Saltiel N, Shah A, Jarnagin J, Baugh CM, Martin BM, Palmisano JN, Cherry JD, Alvarez VE, Huber BR, Weuve J, Nowinski CJ, Cantu RC, Zafonte RD, Dwyer B, Crary JF, Goldstein LE, Kowall NW, Katz DI, Stern RA, Tripodis Y, Stein TD, McClean MD, Alosco ML, McKee AC, Mez J. Leveraging football accelerometer data to quantify associations between repetitive head impacts and chronic traumatic encephalopathy in males. Nat Commun. 2023;14(1):3470. Epub 20230620. doi: 10.1038/s41467-023-39183-0. 37340004 PMC10281995

[8] LeClair J, Weuve J, Fox MP, Mez J, Alosco ML, Nowinski C, McKee A, Tripodis Y. Relationship Between Level of American Football Playing and Diagnosis of Chronic Traumatic Encephalopathy in a Selection Bias Analysis. Am J Epidemiol. 2022;191(8):1429–43. doi: 10.1093/aje/kwac075. 35434739 PMC9989358

[9] Katz DI, Bernick C, Dodick DW, Mez J, Mariani ML, Adler CH, Alosco ML, Balcer LJ, Banks SJ, Barr WB, Brody DL, Cantu RC, Dams-O’Connor K, Geda YE, Jordan BD, McAllister TW, Peskind ER, Petersen RC, Wethe JV, Zafonte RD, Foley É M, Babcock DJ, Koroshetz WJ, Tripodis Y, McKee AC, Shenton ME, Cummings JL, Reiman EM, Stern RA. National Institute of Neurological Disorders and Stroke Consensus Diagnostic Criteria for Traumatic Encephalopathy Syndrome. Neurology. 2021;96(18):848–63. Epub 20210315. doi: 10.1212/wnl.0000000000011850. 33722990 PMC8166432

[10] Montenigro PH, Baugh CM, Daneshvar DH, Mez J, Budson AE, Au R, Katz DI, Cantu RC, Stern RA. Clinical subtypes of chronic traumatic encephalopathy: literature review and proposed research diagnostic criteria for traumatic encephalopathy syndrome. Alzheimers Res Ther. 2014;6(5):68. Epub 20140924. doi: 10.1186/s13195-014-0068-z. 25580160 PMC4288217

[11] Ghajari M, Hellyer PJ, Sharp DJ. Computational modelling of traumatic brain injury predicts the location of chronic traumatic encephalopathy pathology. Brain. 2017;140(2):333–43. Epub 20170102. doi: 10.1093/brain/aww317. 28043957 PMC5278309

[12] Nicks R, Shah A, Stathas SA, Kirsch D, Horowitz SM, Saltiel N, Calderazzo SM, Butler M, Cormier KA, Aytan N, Tu-Zahra F, Mathias R, Faheem F, Marcus S, Spurlock E, Fishbein L, Esnault CD, Boden A, Rosen G, Xia W, Daley S, Meng G, Martin BR, Daneshvar DH, Nowinski CJ, Alosco ML, Mez J, Tripodis Y, Huber BR, Alvarez VE, Cherry JD, McKee AC, Stein TD. Neurodegeneration in the cortical sulcus is a feature of chronic traumatic encephalopathy and associated with repetitive head impacts. Acta Neuropathol. 2024;148(1):79. Epub 20241206. doi: 10.1007/s00401-024-02833-8. 39643767 PMC11624223

[13] Butler M, Dixon E, Stein TD, Alvarez VE, Huber B, Buckland ME, McKee AC, Cherry JD. Tau Pathology in Chronic Traumatic Encephalopathy is Primarily Neuronal. J Neuropathol Exp Neurol. 2022;81(10):773–80. doi: 10.1093/jnen/nlac065. 35903039 PMC9487650

[14] Philibert RA, Beach SR, Brody GH. The DNA methylation signature of smoking: an archetype for the identification of biomarkers for behavioral illness. Nebr Symp Motiv. 2014;61:109–27. doi: 10.1007/978-1-4939-0653-6_6. 25306781 PMC4543297

[15] Rider CF, Carlsten C. Air pollution and DNA methylation: effects of exposure in humans. Clin Epigenetics. 2019;11(1):131. Epub 20190903. doi: 10.1186/s13148-019-0713-2. 31481107 PMC6724236

[16] Koopmans Y, Nelemans SA, Bosmans G, Van Den Noortgate W, Van Leeuwen K, Goossens L. Perceived Parental Support and Psychological Control, DNA Methylation, and Loneliness: Longitudinal Associations Across Early Adolescence. J Youth Adolesc. 2023;52(10):1995–2011. Epub 20230720. doi: 10.1007/s10964-023-01822-6. 37470939

[17] Peng H, Zhu Y, Strachan E, Fowler E, Bacus T, Roy-Byrne P, Goldberg J, Vaccarino V, Zhao J. Childhood Trauma, DNA Methylation of Stress-Related Genes, and Depression: Findings From Two Monozygotic Twin Studies. Psychosom Med. 2018;80(7):599–608. doi: 10.1097/psy.0000000000000604. 29781947 PMC6113110

[18] Haghighi F, Ge Y, Chen S, Xin Y, Umali MU, De Gasperi R, Gama Sosa MA, Ahlers ST, Elder GA. Neuronal DNA Methylation Profiling of Blast-Related Traumatic Brain Injury. J Neurotrauma. 2015;32(16):1200–9. Epub 20150507. doi: 10.1089/neu.2014.3640. 25594545 PMC4532898

[19] Abu Hamdeh S, Ciuculete DM, Sarkisyan D, Bakalkin G, Ingelsson M, Schiöth HB, Marklund N. Differential DNA Methylation of the Genes for Amyloid Precursor Protein, Tau, and Neurofilaments in Human Traumatic Brain Injury. J Neurotrauma. 2021;38(12):1679–88. Epub 20210108. doi: 10.1089/neu.2020.7283. 33191850

[20] Hwang JY, Aromolaran KA, Zukin RS. The emerging field of epigenetics in neurodegeneration and neuroprotection. Nat Rev Neurosci. 2017;18(6):347–61. doi: 10.1038/nrn.2017.46. 28515491 PMC6380351

[21] Jakovcevski M, Akbarian S. Epigenetic mechanisms in neurological disease. Nat Med. 2012;18(8):1194–204. doi: 10.1038/nm.2828. 22869198 PMC3596876

[22] Kochmanski J, Kuhn NC, Bernstein AI. Parkinson’s disease-associated, sex-specific changes in DNA methylation at PARK7 (DJ-1), SLC17A6 (VGLUT2), PTPRN2 (IA-2β), and NR4A2 (NURR1) in cortical neurons. npj Parkinson’s Disease. 2022;8(1):120. doi: 10.1038/s41531-022-00355-2. PMC9508164 36151217

[23] Montalvo-Ortiz JL, Gelernter J, Cheng Z, Girgenti MJ, Xu K, Zhang X, Gopalan S, Zhou H, Duman RS, Southwick SM, Krystal JH, Friedman MJ, Duman RS, Girgenti MJ, Krystal JH, Montalvo-Ortiz JL, Pietrzak RH, Traumatic Stress Brain Research Study G. Epigenome-wide association study of posttraumatic stress disorder identifies novel loci in U.S. military veterans. Translational Psychiatry. 2022;12(1):65. doi: 10.1038/s41398-022-01822-3. 35177594 PMC8854688

[24] Qazi TJ, Quan Z, Mir A, Qing H. Epigenetics in Alzheimer’s Disease: Perspective of DNA Methylation. Mol Neurobiol. 2018;55(2):1026–44. Epub 20170114. doi: 10.1007/s12035-016-0357-6. 28092081

[25] Shireby G, Dempster EL, Policicchio S, Smith RG, Pishva E, Chioza B, Davies JP, Burrage J, Lunnon K, Seiler Vellame D, Love S, Thomas A, Brookes K, Morgan K, Francis P, Hannon E, Mill J. DNA methylation signatures of Alzheimer’s disease neuropathology in the cortex are primarily driven by variation in non-neuronal cell-types. Nature Communications. 2022;13(1):5620. doi: 10.1038/s41467-022-33394-7. PMC9509387 36153390

[26] Mahmood SS, Levy D, Vasan RS, Wang TJ. The Framingham Heart Study and the epidemiology of cardiovascular disease: a historical perspective. Lancet. 2014;383(9921):999–1008. Epub 20130929. doi: 10.1016/s0140-6736(13)61752-3. 24084292 PMC4159698

[27] Mez J, Solomon TM, Daneshvar DH, Murphy L, Kiernan PT, Montenigro PH, Kriegel J, Abdolmohammadi B, Fry B, Babcock KJ, Adams JW, Bourlas AP, Papadopoulos Z, McHale L, Ardaugh BM, Martin BR, Dixon D, Nowinski CJ, Chaisson C, Alvarez VE, Tripodis Y, Stein TD, Goldstein LE, Katz DI, Kowall NW, Cantu RC, Stern RA, McKee AC. Assessing clinicopathological correlation in chronic traumatic encephalopathy: rationale and methods for the UNITE study. Alzheimers Res Ther. 2015;7(1):62. Epub 20151012. doi: 10.1186/s13195-015-0148-8. 26455775 PMC4601147

[28] Hansen KD, Langmead B, Irizarry RA. BSmooth: from whole genome bisulfite sequencing reads to differentially methylated regions. Genome Biol. 2012;13(10):R83. Epub 20121003. doi: 10.1186/gb-2012-13-10-r83. 23034175 PMC3491411

[29] Kolberg L, Raudvere U, Kuzmin I, Vilo J, Peterson H. gprofiler2 -- an R package for gene list functional enrichment analysis and namespace conversion toolset g:Profiler. F1000Res. 2020;9. Epub 20200715. doi: 10.12688/f1000research.24956.2. PMC7859841 33564394

[30] Nicole O, Pacary E. CaMKIIβ in Neuronal Development and Plasticity: An Emerging Candidate in Brain Diseases. Int J Mol Sci. 2020;21(19). Epub 20201001. doi: 10.3390/ijms21197272. PMC7582470 33019657

[31] Mayo L, Trauger SA, Blain M, Nadeau M, Patel B, Alvarez JI, Mascanfroni ID, Yeste A, Kivisäkk P, Kallas K, Ellezam B, Bakshi R, Prat A, Antel JP, Weiner HL, Quintana FJ. Regulation of astrocyte activation by glycolipids drives chronic CNS inflammation. Nat Med. 2014;20(10):1147–56. Epub 20140914. doi: 10.1038/nm.3681. 25216636 PMC4255949

[32] Deans MR, Krol A, Abraira VE, Copley CO, Tucker AF, Goodrich LV. Control of neuronal morphology by the atypical cadherin Fat3. Neuron. 2011;71(5):820–32. doi: 10.1016/j.neuron.2011.06.026. 21903076 PMC3521586

[33] Ramos AD, Andersen RE, Liu SJ, Nowakowski TJ, Hong SJ, Gertz C, Salinas RD, Zarabi H, Kriegstein AR, Lim DA. The long noncoding RNA Pnky regulates neuronal differentiation of embryonic and postnatal neural stem cells. Cell Stem Cell. 2015;16(4):439–47. Epub 20150319. doi: 10.1016/j.stem.2015.02.007. 25800779 PMC4388801

[34] Sorokina EA, Reis LM, Thompson S, Agre K, Babovic-Vuksanovic D, Ellingson MS, Hasadsri L, van Bever Y, Semina EV. WDR37 syndrome: identification of a distinct new cluster of disease-associated variants and functional analyses of mutant proteins. Hum Genet. 2021;140(12):1775–89. Epub 20211012. doi: 10.1007/s00439-021-02384-y. 34642815 PMC9241141

[35] Shigemizu D, Akiyama S, Mitsumori R, Niida S, Ozaki K. Identification of potential blood biomarkers for early diagnosis of Alzheimer’s disease through immune landscape analysis. NPJ Aging. 2022;8(1):15. Epub 20221104. doi: 10.1038/s41514-022-00096-9. 36333348 PMC9636153

[36] Gambuzza ME, Sofo V, Salmeri FM, Soraci L, Marino S, Bramanti P. Toll-like receptors in Alzheimer’s disease: a therapeutic perspective. CNS Neurol Disord Drug Targets. 2014;13(9):1542–58. doi: 10.2174/1871527313666140806124850. 25106635

[37] Hayward JH, Lee SJ. A Decade of Research on TLR2 Discovering Its Pivotal Role in Glial Activation and Neuroinflammation in Neurodegenerative Diseases. Exp Neurobiol. 2014;23(2):138–47. Epub 20140613. doi: 10.5607/en.2014.23.2.138. 24963278 PMC4065827

[38] Iffland PH, Everett ME, Cobb-Pitstick KM, Bowser LE, Barnes AE, Babus JK, Romanowski AJ, Baybis M, Elziny S, Puffenberger EG, Gonzaga-Jauregui C, Poulopoulos A, Carson VJ, Crino PB. NPRL3 loss alters neuronal morphology, mTOR localization, cortical lamination and seizure threshold. Brain. 2022;145(11):3872–85. doi: 10.1093/brain/awac044. 35136953 PMC10200289

[39] Nakamura K, Mori F, Tanji K, Miki Y, Yamada M, Kakita A, Takahashi H, Utsumi J, Sasaki H, Wakabayashi K. Isopentenyl diphosphate isomerase, a cholesterol synthesizing enzyme, is localized in Lewy bodies. Neuropathology. 2015;35(5):432–40. Epub 20150507. doi: 10.1111/neup.12204. 25950736

[40] Ohara-Imaizumi M, Yoshida M, Aoyagi K, Saito T, Okamura T, Takenaka H, Akimoto Y, Nakamichi Y, Takanashi-Yanobu R, Nishiwaki C, Kawakami H, Kato N, Hisanaga S, Kakei M, Nagamatsu S. Deletion of CDKAL1 affects mitochondrial ATP generation and first-phase insulin exocytosis. PLoS One. 2010;5(12):e15553. Epub 20101209. doi: 10.1371/journal.pone.0015553. 21151568 PMC3000340

[41] Di G, Yang X, Cheng F, Liu H, Xu M. CEBPA-AS1 Knockdown Alleviates Oxygen-Glucose Deprivation/Reperfusion-Induced Neuron Cell Damage by the MicroRNA 24–3p/BOK Axis. Mol Cell Biol. 2021;41(8):e0006521. Epub 20210723. doi: 10.1128/mcb.00065-21. 34001648 PMC8300783

[42] Al-Naama N, Mackeh R, Kino T. C(2)H(2)-Type Zinc Finger Proteins in Brain Development, Neurodevelopmental, and Other Neuropsychiatric Disorders: Systematic Literature-Based Analysis. Front Neurol. 2020;11:32. Epub 20200214. doi: 10.3389/fneur.2020.00032. 32117005 PMC7034409

[43] Cubillos-Rojas M, Schneider T, Hadjebi O, Pedrazza L, de Oliveira JR, Langa F, Guénet JL, Duran J, de Anta JM, Alcántara S, Ruiz R, Pérez-Villegas EM, Aguilar-Montilla FJ, Carrión Á M, Armengol JA, Baple E, Crosby AH, Bartrons R, Ventura F, Rosa JL. The HERC2 ubiquitin ligase is essential for embryonic development and regulates motor coordination. Oncotarget. 2016;7(35):56083–106. doi: 10.18632/oncotarget.11270. 27528230 PMC5302898

[44] Benesh AE, Fleming JT, Chiang C, Carter BD, Tyska MJ. Expression and localization of myosin-1d in the developing nervous system. Brain Res. 2012;1440:9–22. Epub 20120108. doi: 10.1016/j.brainres.2011.12.054. 22284616 PMC3278530

[45] Rizzardi LF, Hickey PF, Rodriguez DiBlasi V, Tryggvadóttir R, Callahan CM, Idrizi A, Hansen KD, Feinberg AP. Neuronal brain-region-specific DNA methylation and chromatin accessibility are associated with neuropsychiatric trait heritability. Nature Neuroscience. 2019;22(2):307–16. doi: 10.1038/s41593-018-0297-8. 30643296 PMC6348048

[46] Iwamoto K, Bundo M, Ueda J, Oldham MC, Ukai W, Hashimoto E, Saito T, Geschwind DH, Kato T. Neurons show distinctive DNA methylation profile and higher interindividual variations compared with non-neurons. Genome Res. 2011;21(5):688–96. Epub 20110405. doi: 10.1101/gr.112755.110. 21467265 PMC3083085

[47] Kerwin J, Yücesoy A, Vidhate S, Dávila-Montero BM, Van Orman JL, Pence TJ, Tartis M, Mejía-Alvarez R, Willis AM. Sulcal Cavitation in Linear Head Acceleration: Possible Correlation With Chronic Traumatic Encephalopathy. Front Neurol. 2022;13:832370. Epub 20220228. doi: 10.3389/fneur.2022.832370. 35295830 PMC8918564

[48] Butler MLMD, Pervaiz N, Breen K, Calderazzo S, Ypsilantis P, Wang Y, Breda JC, Mazzilli S, Nicks R, Spurlock E, Hefti MM, Fiock KL, Huber BR, Alvarez VE, Stein TD, Campbell JD, McKee AC, Cherry JD. Repeated head trauma causes neuron loss and inflammation in young athletes. Nature. 2025. doi: 10.1038/s41586-025-09534-6. PMC12589125 40963024

[49] Kool MJ, Proietti Onori M, Borgesius NZ, van de Bree JE, Elgersma-Hooisma M, Nio E, Bezstarosti K, Buitendijk GHS, Aghadavoud Jolfaei M, Demmers JAA, Elgersma Y, van Woerden GM. CAMK2-Dependent Signaling in Neurons Is Essential for Survival. J Neurosci. 2019;39(28):5424–39. Epub 20190507. doi: 10.1523/jneurosci.1341-18.2019. 31064859 PMC6616294

[50] Zou J, Lei C, Zhang Y, Ma A, Meng Z, Zhu J, Lin H, Zhang G, Liang Y, Tan M. CaMKIIβ-mediated Phosphorylation Enhances Protein Stability of Spastin to Promote Neurite Outgrowth. J Neurosci. 2025;45(32). Epub 20250806. doi: 10.1523/jneurosci.1995-24.2025. PMC12330322 40645772

[51] Borghi R, Trivisano M, Specchio N, Tartaglia M, Compagnucci C. Understanding the pathogenetic mechanisms underlying altered neuronal function associated with CAMK2B mutations. Neurosci Biobehav Rev. 2023;152:105299. Epub 20230628. doi: 10.1016/j.neubiorev.2023.105299. 37391113

[52] Yang C, Zhang M, Li S, Yi F, Huang H, Xie H, Liu H, Huang R, Zhou J. Effects of Camk2b Overexpression and Underexpression on the Proteome of Rat Hippocampal Neurons. Neuroscience. 2022;503:58–68. Epub 20220827. doi: 10.1016/j.neuroscience.2022.08.019. 36041587

[53] Ghosh A, Giese KP. Calcium/calmodulin-dependent kinase II and Alzheimer’s disease. Molecular Brain. 2015;8(1):78. doi: 10.1186/s13041-015-0166-2. 26603284 PMC4657223

[54] Zhang GR, Cheng XR, Zhou WX, Zhang YX. Age-related expression of calcium/calmodulin-dependent protein kinase II A in the hippocampus and cerebral cortex of senescence accelerated mouse prone/8 mice is modulated by anti-Alzheimer’s disease drugs. Neuroscience. 2009;159(1):308–15. Epub 20080725. doi: 10.1016/j.neuroscience.2008.06.068. 18721865

[55] Habif M, Do Carmo S, Báez MV, Colettis NC, Cercato MC, Salas DA, Acutain MF, Sister CL, Berkowicz VL, Canal MP, González Garello T, Cuello AC, Jerusalinsky DA. Early Long-Term Memory Impairment and Changes in the Expression of Synaptic Plasticity-Associated Genes, in the McGill-R-Thy1-APP Rat Model of Alzheimer’s-Like Brain Amyloidosis. Front Aging Neurosci. 2020;12:585873. Epub 20210122. doi: 10.3389/fnagi.2020.585873. 33551786 PMC7862771

[56] Borgesius NZ, van Woerden GM, Buitendijk GH, Keijzer N, Jaarsma D, Hoogenraad CC, Elgersma Y. βCaMKII plays a nonenzymatic role in hippocampal synaptic plasticity and learning by targeting αCaMKII to synapses. J Neurosci. 2011;31(28):10141–8. doi: 10.1523/jneurosci.5105-10.2011. 21752990 PMC6623068

[57] Estades Ayuso V, Pickles S, Todd T, Yue M, Jansen-West K, Song Y, González Bejarano J, Rawlinson B, DeTure M, Graff-Radford NR, Boeve BF, Knopman DS, Petersen RC, Dickson DW, Josephs KA, Petrucelli L, Prudencio M. TDP-43-regulated cryptic RNAs accumulate in Alzheimer’s disease brains. Mol Neurodegener. 2023;18(1):57. Epub 20230821. doi: 10.1186/s13024-023-00646-z. 37605276 PMC10441763

[58] Chung M, Carter EK, Veire AM, Dammer EB, Chang J, Duong DM, Raj N, Bassell GJ, Glass JD, Gendron TF, Nelson PT, Levey AI, Seyfried NT, McEachin ZT. Cryptic exon inclusion is a molecular signature of LATE-NC in aging brains. Acta Neuropathol. 2024;147(1):29. Epub 20240203. doi: 10.1007/s00401-023-02671-0. 38308693 PMC10838224

[59] Trautwig AN, Shantaraman A, Chung M, Dammer EB, Ping L, Duong DM, Petrucelli L, Ward ME, Glass JD, Nelson PT, Levey AI, McEachin ZT, Seyfried NT. Molecular subtyping based on hippocampal cryptic exon burden reveals proteome-wide changes associated with TDP-43 pathology across the spectrum of LATE and Alzheimer’s Disease. bioRxiv. 2025. Epub 20250702. doi: 10.1101/2025.05.30.656396.

[60] Yoshihara T, Satake H, Nishie T, Okino N, Hatta T, Otani H, Naruse C, Suzuki H, Sugihara K, Kamimura E, Tokuda N, Furukawa K, Fururkawa K, Ito M, Asano M. Lactosylceramide synthases encoded by B4galt5 and 6 genes are pivotal for neuronal generation and myelin formation in mice. PLoS Genet. 2018;14(8):e1007545. Epub 20180816. doi: 10.1371/journal.pgen.1007545. 30114188 PMC6095488

[61] Tokuda N, Numata S, Li X, Nomura T, Takizawa M, Kondo Y, Yamashita Y, Hashimoto N, Kiyono T, Urano T, Furukawa K, Furukawa K. β4GalT6 is involved in the synthesis of lactosylceramide with less intensity than β4GalT5. Glycobiology. 2013;23(10):1175–83. Epub 20130723. doi: 10.1093/glycob/cwt054. 23882130

