# Supplementary material for: Duration of contact sports play associated with aberrant DNA methylation in human frontal cortex
Source: Res Sq. 2026 Jan 7:rs.3.rs-7861173. Originally published 2025 Dec 1. Preprint. [Version 2] doi: 10.21203/rs.3.rs-7861173/v2 (PMC12687817; doi:10.21203/rs.3.rs-7861173/v2)
Supplement: Supplement 1 [file NIHPPRS7861173V2-supplement-1.pdf]

Supplementary Files

This is a list of supplementary files associated with this preprint. Click to download.

- SupplementaryTable1.xlsx
- SupplementaryTable2.xlsx

- SupplementaryTable3.xlsx
